# Extracts of *Aspidopterys tomentosa* Attenuate Nephrolithiasis via Inhibiting Endoplasmic Reticulum Stress

**DOI:** 10.3390/ph19071049

**Published:** 2026-07-07

**Authors:** Shifang Liu, Meng Li, Jing Yu, Cuiyun Yin, Siqi Li, Zhaoyou Deng, Yin Yuan, Xuanchao Shi, Deying Tang, Yihang Li, Xi Chen

**Affiliations:** 1 Yunnan Key Laboratory of Southern Medicine Utilization, Yunnan Branch of Institute of Medicinal Plant Development, Chinese Academy of Medical Sciences & Peking Union Medical College, Jinghong 666100, China; sfliu@implad.ac.cn (S.L.); yujingynu1113@163.com (J.Y.); cyyin@implad.ac.cn (C.Y.); pharmacologyvip@163.com (Z.D.); swyuanyin@126.com (Y.Y.); xcs5977@163.com (X.S.); tdy629@126.com (D.T.); 2 School of Chemistry and Environment, Yunnan Minzu University, 2929 Yuehua Street, Chenggong District, Kunming 650504, China; 18213448472@163.com; 3 Xishuangbanna Dai Medical Hospital, Jinghong 666100, China; l1868193928@163.com

**Keywords:** calcium oxalate nephrolithiasis, *Aspidopterys tomentosa*, endoplasmic reticulum stress, steroidal saponins

## Abstract

**Objectives**: *Aspidopterys obcordata* has been traditionally used by the Dai people in Xishuangbanna, China, for the prevention and treatment of renal calculi. This study aimed to investigate the inhibitory effect of *A. tomentosa* extracts on calcium oxalate stone formation. **Methods**: The extracts of *A. tomentosa* (EA) were obtained via 95% ethanol reflux extraction, followed by multi-polar solvent extraction and elution. The HK-2 cell injury model induced by calcium oxalate and the renal calculus mouse model established by intraperitoneal injection of glyoxylic acid were established to assess drug efficacy. EA intervention was performed to evaluate its effects on calcium oxalate crystal deposition, renal tubular injury, cell apoptosis, and serum creatinine (Scr) and blood urea nitrogen (BUN) levels. Furthermore, the potential mechanism underlying, particularly the regulation of PERK/ATF4/CHOP signaling pathway and endoplasmic reticulum stress-mediated apoptosis, was investigated. **Results**: EA treatment significantly reduced renal calcium oxalate crystal deposition, alleviated renal tubular injury, inhibited cell apoptosis, and decreased Scr and BUN levels. Mechanistically, the protective effects of EA were mediated by the downregulation of the PERK/ATF4/CHOP signaling pathway and the suppression of endoplasmic reticulum stress-mediated apoptosis. **Conclusions**: These findings provide experimental evidence supporting that *A. tomentosa* can be developed as a promising agent for the prevention of nephrolithiasis.

## 1. Introduction

Calcium oxalate (CaOx) nephrolithiasis is one of the most prevalent urinary system disorders, with a steadily rising global incidence closely linked to metabolic syndrome, dietary patterns, and other risk factors [1,2,3]. Beyond inducing severe renal colic, hematuria, and urinary tract infections, poorly managed nephrolithiasis may progress to renal dysfunction, thereby substantially impairing patients’ quality of life [4,5,6,7]. Although minimally invasive lithotripsy and pharmacological interventions for nephrolithiasis are clinically mature, they cannot prevent renal stone recurrence. The postoperative recurrence rate of nephrolithiasis is 40–50% within five years and reaches up to 50–60% within ten years after treatment [8]. Therefore, it is imperative to develop targeted preventive strategies for both new and recurrent renal stones to alleviate the physical and economic burdens caused by nephrolithiasis [9]. Currently, clinically available drugs for nephrolithiasis prevention are limited in efficacy and accompanied by obvious side effects. Therefore, exploring highly effective and low-toxicity anti-nephrolithiasis active ingredients from natural traditional Chinese medicines has become a key focus and research direction in the field of urinary system diseases [10,11,12,13].

*Aspidopterys tomentosa* var. Tomentosa, formerly named *Aspidopterys obcordata* Hemsl., is a traditional Dai medicinal herb native to China. It has long been clinically applied for the treatment of renal calculi and the prevention of post-lithotripsy stone recurrence with satisfactory therapeutic efficacy [14,15,16]. Although previous studies have demonstrated that steroidal saponins derived from *A. tomentosa* can alleviate calcium oxalate crystal-induced renal tubular epithelial cell injury and inhibit stone formation, the pharmacodynamic material basis and core molecular mechanisms responsible for its anti-nephrolithiasis effects remain unclear, which greatly limits the in-depth development and clinical translation of this valuable ethnic medicinal resource [15,17,18,19].

Accordingly, steroidal saponins from *A. tomentosa* were isolated, purified and enriched in this study. Subsequently, a calcium oxalate monohydrate (COM) crystal-induced renal tubular epithelial cell injury model and a glyoxylate-induced mouse nephrolithiasis model were established to evaluate the preventive effect of these saponin-enriched extracts on nephrolithiasis, thereby providing a fundamental basis for exploring their druggability.

## 2. Results

### 2.1. Results of Chemical Composition Analysis of the Sample

**Figure 1:**
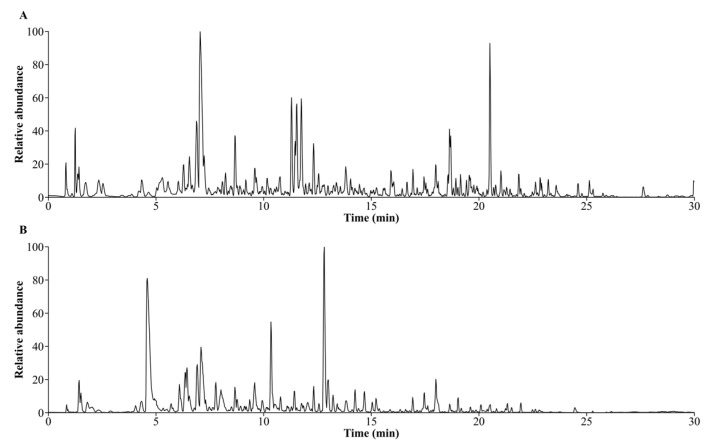
EA detection base peak chromatogram. ((**A**): positive ion mode, (**B**): negative ion mode).

The steroidal saponin content of the prepared sample was determined to be approximately 40.11% using UV spectrophotometry (Figure S1). Subsequent UHPLC-Q-Orbitrap MS analysis, combined with database matching against TCM Pro 2.0 and theoretical databases, unambiguously identified 21 compounds in the steroidal saponin fraction (Figure 1, Table 1).

### 2.2. EA Attenuates COM-Induced Injury in HK-2 Cells

**Figure 2:**
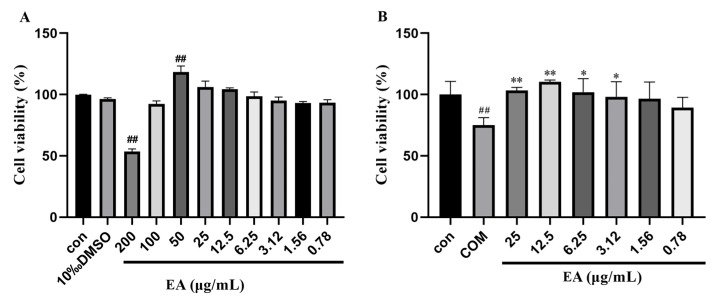
Effect of COM crystals on cell viability (**A**); Effect of EA on COM crystal-induced changes in cell viability (**B**). Note: Compared with the con group, ^##^ indicates *p* < 0.01; compared with the COM group, * indicates *p* < 0.05, ** indicates *p* < 0.01.

EA exerted no significant cytotoxicity against HK-2 cells at concentrations ranging from 0.78 to 25 μg/mL (Figure 2A). Compared with the control group, cell viability was markedly decreased in the COM-treated model group. Pretreatment with EA 3.13, 6.25, 12.5, and 25 μg/mL significantly restored cell viability in a dose-dependent manner, with differences relative to the model group (Figure 2B).

### 2.3. EA Reduces ROS Levels and Inhibits Apoptosis in COM-Damaged HK-2 Cells

**Figure 3:**
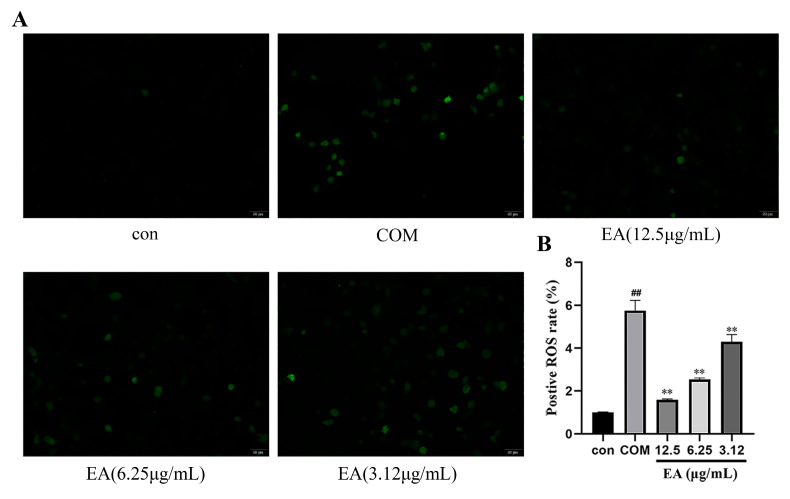
EA attenuates ROS generation in HK-2 cells. (**A**) DCFH-DA staining images of HK-2 cells in the COM-induced injury model after EA intervention. (**B**) DCFH-DA Staining Positivity of HK-2 cells in the COM-induced injury model after EA intervention (*n* = 6). Note: Compared with the con group, ^##^ indicates *p* < 0.01; compared with the COM group, ** indicates *p* < 0.01.

COM crystals significantly increased intracellular reactive oxygen species (ROS) levels in HK-2 cells compared with the control group. EA treatment suppressed COM-induced ROS overproduction in a dose-dependent manner, and this inhibitory effect was positively correlated with EA concentration (Figure 3).

**Figure 4:**
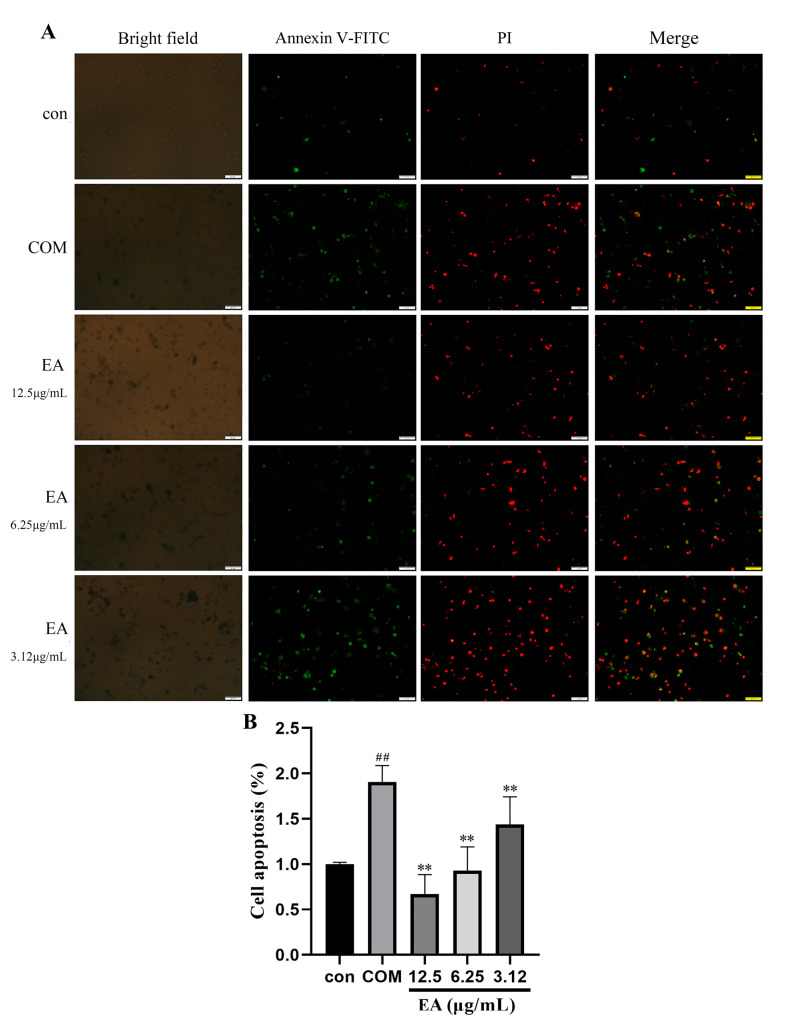
EA reduced the COM crystal-induced apoptosis and necrosis of HK-2 cells. (**A**) Representative images of Annexin V-FITC and PI staining in HK-2 cells subjected to COM-induced injury following EA intervention. (**B**) Apoptosis rate of HK-2 cells in the COM-induced injury model after EA intervention (*n* = 3). Note: Compared with the con group, ^##^ indicates *p* < 0.01; Compared with the COM group, ** indicates *p* < 0.01.

Annexin V-PI staining yielded consistent apoptotic results. The number of apoptotic HK-2 cells was markedly higher in the COM group than in the control group. EA treatment significantly reduced COM-induced cell apoptosis (Figure 4).

### 2.4. EA Reduces BUN and Scr Levels in Mice with Nephrolithiasis

**Figure 5:**
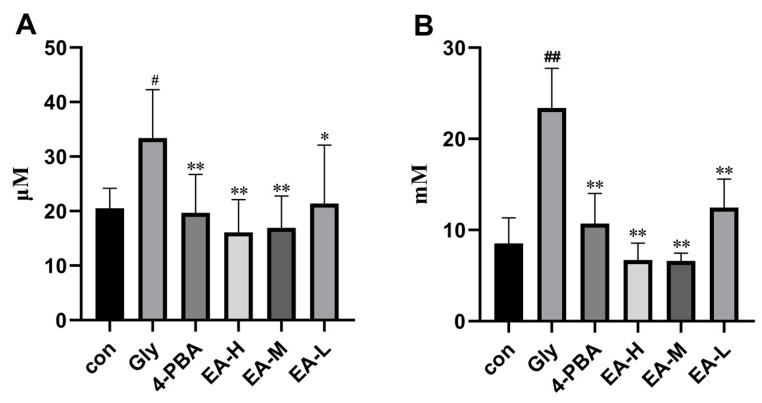
EA exerts a protective effect against Gly-induced renal function injury in mice. (**A**) EA decreased Scr levels in the nephrolithiasis model. (**B**) EA decreased BUN levels in the nephrolithiasis mice (n = 8). Note: Compared with the con group, ^#^ indicates *p* < 0.05, ^##^ indicates *p* < 0.01; compared with the Gly group, * indicates *p* < 0.05, ** indicates *p* < 0.01.

Compared with the normal control group, the levels of BUN and Scr in the model group were significantly increased. After treatment with low-, medium-, and high-dose EA as well as 4-PBA, BUN and Scr levels were markedly decreased (Figure 5). These results indicate that glyoxylate (Gly) induction causes significant renal dysfunction in mice, and EA exerts a protective effect against such renal injury.

### 2.5. EA Alleviates Renal Pathological Injury and Apoptosis In Vivo

**Figure 6:**
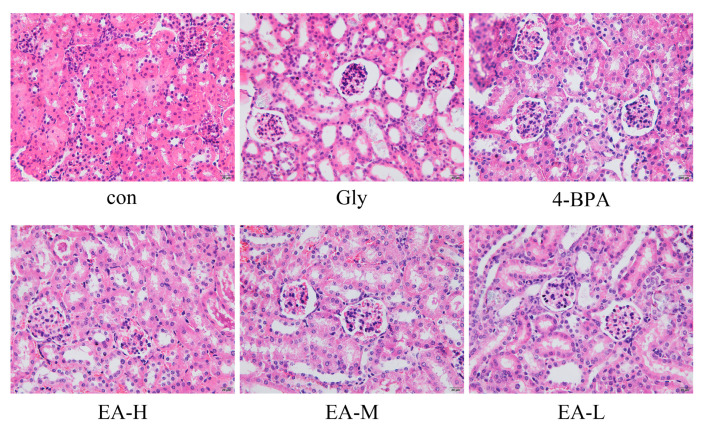
EA alleviates renal pathological damage in the nephrolithiasis model mice (400×).

Mice in the control group exhibited normal glomerular architecture, clear tubular contours, tightly arranged renal tissues, and no visible crystal deposition. In the Gly group, numerous large calcium oxalate crystals were extensively distributed in the renal cortex and medulla, mainly accumulating in the renal tubules, accompanied by a loose tissue structure and obvious tubular dilation. Compared with the Gly group, renal crystal deposition was significantly reduced in EA-treated mice. Notably, no obvious calcium oxalate crystals were observed in the medium-dose EA, high-dose EA, and 4-PBA group. Moreover, the pathological damage to glomeruli and renal tubules was markedly ameliorated in the high-dose EA group, whereas only small and sporadic calcium oxalate crystals were occasionally detected in the low-dose EA group (Figure 6). Collectively, these results indicate that EA effectively alleviates Gly-induced renal injury and reduces calcium oxalate crystal deposition in mice.

TUNEL fluorescence staining yielded consistent trends. Compared with the control group, the number of apoptotic cells in renal tissues was significantly increased in the model group. After EA intervention, the number of apoptotic cells was markedly reduced in a dose-dependent manner relative to the model group (Figure S2A). The variation trend observed in vivo was consistent with that in COM-induced HK-2 cell models (Figure S2B).

### 2.6. EA Downregulates the PERK/ATF4/CHOP Signaling Pathway and Apoptotic Protein Expression in HK-2 Cells and Mouse Renal Tissues

**Figure 7:**
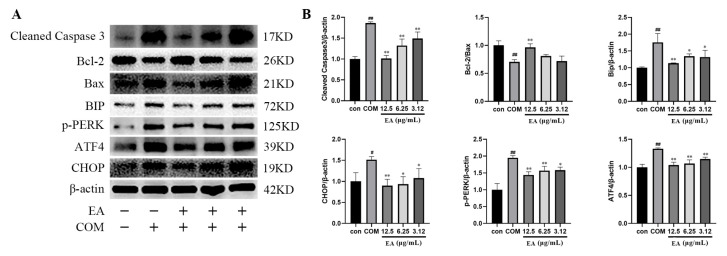
EA reverses the upregulated expression of apoptotic proteins in COM crystal-stimulated HK-2 cells. (**A**) Representative Western blotting images of endoplasmic reticulum stress and apoptosis-related proteins in HK-2 cells; (**B**) Expression levels of endoplasmic reticulum stress and apoptosis-related proteins in HK-2 cells (n = 3). Note: Compared with the con group, ^#^ indicates *p* < 0.05, ^##^ indicates *p* < 0.01; compared with the COM group, * indicates *p* < 0.05, ** indicates *p* < 0.01.

EA significantly inhibited the COM crystal-induced upregulation of Cleaved-caspase 3 expression and increased the Bcl-2/Bax ratio in a dose-dependent manner, thereby suppressing the expression of apoptotic proteins. The expression levels of endoplasmic reticulum stress (ERS) marker proteins CHOP and Bip were markedly elevated upon COM crystal stimulation, whereas EA treatment effectively reversed these alterations. These results indicated that EA alleviated COM crystal-triggered ERS injury (Figure 7). Furthermore, EA inhibited PERK phosphorylation and downregulated ATF4 expression, indicating that EA suppresses the activation of the PERK/ATF4/CHOP signaling and blocks ERS-mediated apoptosis induced by COM crystals.

**Figure 8:**
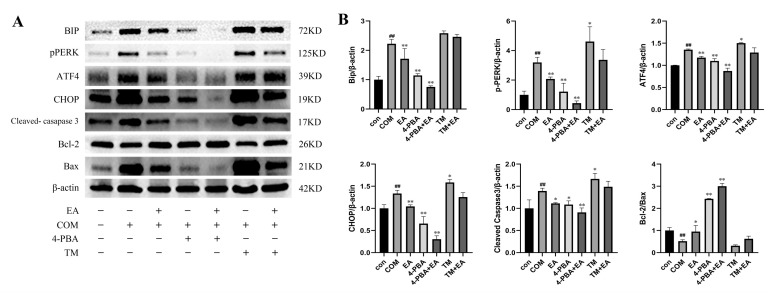
Effects of ER inhibitor 4-PBA, agonist TM on COM-induced ERS and apoptosis in HK-2 cells. (**A**) Representative Western blotting images of endoplasmic reticulum stress and apoptosis-related proteins in HK-2 cells; (**B**) Expression levels of endoplasmic reticulum stress and apoptosis-related proteins in HK-2 cells (n = 3). Note: Compared with the con group, ^##^ indicates *p* < 0.01; compared with the COM group, * indicates *p* < 0.05, ** indicates *p* < 0.01.

To further verify the regulatory effect of EA on the PERK/ATF4/CHOP signaling pathway, TM and 4-PBA were used as ERS agonists and inhibitors for intervention, respectively. As shown in Figure 8, 4-PBA significantly downregulated the COM crystal-induced upregulation of ERS-related proteins (BIP, p-PERK, ATF4, CHOP) and the apoptotic protein Cleaved-caspase 3, while increasing the Bcl-2/Bax ratio in HK-2 cells. In contrast, TM further promoted the expression of these related proteins in COM crystal-induced HK-2 cells. Combined treatment with 4-PBA and EA exerted enhanced inhibitory effects on ERS and apoptosis. Notably, EA reversed the TM-mediated upregulation of ERS and apoptotic protein expression, restoring these indicators to the levels observed in the sole COM treatment group. These findings further confirm that EA exerts protective effects against COM crystal-induced injury in HK-2 cells via modulating the PERK/ATF4/CHOP-mediated ERS apoptotic pathway.

**Figure 9:**
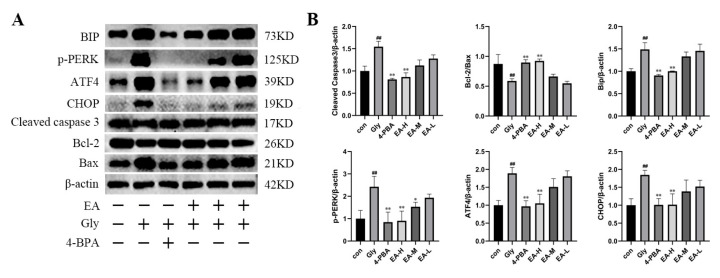
EA reduced the expression of endoplasmic reticulum stress and apoptosis-related proteins in the kidneys of the nephrolithiasis model mice. (**A**) Representative Western blotting images of endoplasmic reticulum stress and apoptosis-related proteins in kidneys; (**B**) Expression levels of endoplasmic reticulum stress and apoptosis-related proteins in kidneys (n = 3). Note: Compared with the con group, ^##^ indicates *p* < 0.01; compared with the Gly group, * indicates *p* < 0.05, ** indicates *p* < 0.01.

In the Gly-induced mice nephrolithiasis model, high-dose EA effectively inhibited the expression of the key apoptotic protein cleaved-caspase 3 and elevated the anti-apoptotic Bcl-2/Bax ratio. Although medium- and low-dose EA did not produce statistically significant differences in this index, an obvious upward trend was observed. As shown in Figure 9, glyoxylate activated the ERS-related PERK signaling pathway in mouse renal tissues, markedly upregulating the expression of BIP and CHOP and increasing PERK phosphorylation and ATF4 protein levels. In contrast, intervention with high-dose EA or 4-PBA effectively blocked the activation of this pathway and significantly downregulated the expression of ERS-related proteins. These results further confirm that EA alleviates glyoxylate-induced ERS by modulating the PERK/ATF4/CHOP signaling pathway.

## 3. Discussion

Calcium oxalate nephrolithiasis arises from a complex interplay of multiple pathogenic factors, yet its exact molecular and pathological mechanisms remain poorly understood. Emerging studies have confirmed that urinary supersaturation with calcium oxalate crystals alone cannot drive lithogenesis, and pathological dysfunction of renal tubular epithelial cells serves as a critical contributor to stone formation [20]. Despite the presence of physiological urinary supersaturation and calcium oxalate crystallization in healthy populations, intact renal tubular epithelial cells possess inherent self-protective capacities and anti-adhesion properties against crystals under normal physiological conditions. Such biological characteristics effectively prevent heterogeneous nucleation and other essential lithogenic processes on the cell surface [21]. Specifically, damage and dysfunction of renal tubular epithelial cells can form initial pathological nidi, which constitute the vital initiating event for the occurrence and recurrence of nephrolithiasis [22]. Therefore, alleviating renal tubular epithelial cell injury is a key therapeutic strategy to inhibit the recurrence of calcium oxalate kidney stones. Our previous study demonstrated that steroidal saponins from *A. tomentosa* protect renal tubular epithelial cells against calcium oxalate crystal-induced injury, which is considered the key mechanism responsible for its preventive effect on nephrolithiasis [17]. Accordingly, we prepared steroidal saponin-enriched extracts and carried out pharmacological studies.

Renal tubular epithelial cell injury serves as a core pathological mechanism underlying the initiation and progression of CaOx nephrolithiasis, which is tightly correlated with ERS induced by disrupted endoplasmic reticulum homeostasis [23,24]. CaOx crystals directly target renal tubular epithelial cells and trigger intracellular oxidative stress imbalance and protein-folding dysfunction, thereby disrupting endoplasmic reticulum homeostasis and initiating sustained, uncontrolled ERS responses. Excessive ERS further activates downstream apoptotic signaling cascades, ultimately inducing renal tubular epithelial cell apoptosis. Damaged and apoptotic renal tubular epithelial cells secrete abundant inflammatory factors and crystal adhesion molecules, which further promote the adhesion, aggregation, and growth of CaOx crystals. This process forms a vicious cycle of “cell injury—crystal deposition—exacerbated injury”, which ultimately accelerates the formation and progression of kidney stones [25,26,27,28]. Numerous studies have verified that the PERK/ATF4/CHOP signaling pathway is a classic regulatory pathway of ERS-mediated apoptosis and plays a critical role in the progression of renal injury diseases [29,30]. When endoplasmic reticulum homeostasis is disrupted, the molecular chaperone glucose-regulated protein 78 (Grp78) dissociates from PERK, thereby inducing PERK autophosphorylation and activation. Activated PERK further phosphorylates eukaryotic translation initiation factor 2α (eIF2α), which facilitates the expression and nuclear translocation of transcription factor ATF4 [31,32]. ATF4 directly binds to the promoter region of the pro-apoptotic CHOP gene, markedly upregulates CHOP expression, and ultimately initiates intracellular apoptotic programs. This cascade exacerbates renal tubular epithelial cell injury and death, thereby driving the pathological progression of CaOx nephrolithiasis [33,34].

In this study, experimental results demonstrated that EA effectively antagonized COM crystal-induced renal tubular epithelial cell injury. EA treatment not only significantly restored the viability of damaged renal tubular epithelial cells and ameliorated crystal stimulation-induced morphological abnormalities, but also markedly decreased the cellular apoptosis rate. These findings indicate that EA exerts prominent protective effects on renal tubular epithelial cells and possesses potent anti-nephrolithiasis activity. To further elucidate the underlying molecular mechanism, the present study focused on the ERS-related PERK/ATF4/CHOP signaling pathway and apoptosis-related proteins. The results revealed that CaOx crystal stimulation significantly upregulated the protein expression levels of phosphorylated PERK (p-PERK), ATF4, and CHOP in renal tubular epithelial cells. Additionally, COM crystal treatment increased the expression of pro-apoptotic proteins Bax and caspase 3, while downregulating the level of anti-apoptotic protein Bcl-2. These alterations indicate abnormal activation of the PERK/ATF4/CHOP pathway and the initiation of cellular apoptotic procedures. After EA intervention, the phosphorylation level of PERK was significantly reduced in renal tubular epithelial cells. Similarly, the protein levels of ATF4 and CHOP were markedly downregulated. EA treatment also effectively suppressed the abnormal overexpression of pro-apoptotic Bax and caspase 3, whereas the level of the anti-apoptotic protein Bcl-2 was significantly upregulated. Collectively, these data demonstrate that EA targets and inhibits the excessive activation of the ERS-dependent PERK/ATF4/CHOP signaling cascade. EA further reverses the imbalance of Bax/cleaved-caspase 3/Bcl-2 expression, blocks ERS-mediated apoptotic signal transduction, and ultimately alleviates COM crystal-induced renal tubular epithelial cell apoptosis. By maintaining the structural integrity and physiological function of renal tubular epithelial cells, EA reduces crystal adhesion and deposition, thereby exerting anti-nephrolithiasis effects.

To further mimic the pathophysiological microenvironment of human nephrolithiasis and validate the in vivo anti-lithic efficacy and underlying molecular mechanism of EA, a Gly-induced renal stone mouse model was established in the present study. The cellular protective effects and therapeutic efficacy of EA were comprehensively verified from three dimensions, including renal function, renal histopathology, and pathway-related protein expression. Renal function detection revealed that compared with normal mice, model mice exhibited significantly elevated Scr and BUN levels, accompanied by severely impaired glomerular filtration and tubular metabolic functions, confirming successful model establishment [35,36,37,38]. After intervention with gradient doses of EA, the abnormal elevation of Scr and BUN in model mice was markedly reversed in a dose-dependent manner. These findings suggested that EA effectively ameliorated stone-induced renal dysfunction, restored renal filtration and metabolic excretion capacity, and exerted prominent systemic renal protective effects. Renal pathological observations further validated the therapeutic effect of EA [39,40,41]. Severe lithic pathological damage was observed in the renal tissues of model mice, characterized by destroyed renal tubular structure, tubular dilation, epithelial vacuolar degeneration and exfoliation, obvious interstitial inflammatory infiltration, and extensive CaOx crystals deposition and aggregation within the tubular lumen. In contrast, EA intervention significantly alleviated renal pathological lesions in model mice. The integrity of renal tubular structure was restored, epithelial vacuolar degeneration and shedding were significantly improved, interstitial inflammatory infiltration was attenuated, and the quantity and volume of CaOx crystal deposits were prominently decreased. These results demonstrated that EA could repair renal tissue morphological damage, inhibit renal crystal deposition and growth, and hinder the pathological progression of nephrolithiasis.

Collectively, this study elucidates partial molecular mechanisms underlying the anti-nephrolithiasis effect of EA against CaOx stone formation from the perspective of ERS-mediated cellular apoptosis. It is verified that EA exerts a preventive effect on nephrolithiasis by specifically inhibiting the excessive activation of the PERK/ATF4/CHOP signaling pathway, which provides an experimental basis for the subsequent development of EA as a novel traditional Chinese medicinal preparation for nephrolithiasis prevention. Nevertheless, the present study has several limitations. The content of steroidal saponins in EA is relatively low, and the extraction, separation, and purification processes of EA require further systematic optimization. In addition, the evaluation of renal pathological sections in this study lacked quantitative renal injury scoring, and CaOx crystal deposition was not strictly quantified. Furthermore, the exploration of the underlying signaling regulatory mechanism relied solely on pharmacological intervention, without genetic validation through gene knockdown or silencing strategies. These limitations in experimental design and mechanistic verification warrant further investigation in future studies.

## 4. Materials and Methods

### 4.1. Chemical Composition Analysis of the Sample

*A. tomentosa* were collected in Jinghong City, Yunnan Province, China, and taxonomically identified by Researcher Tang Deying. Only the main vine stems were used, with other aerial tissues discarded. The plant materials were dried and processed for subsequent experiments.

Dried stems of *A. tomentosa* (5 kg) were ground into coarse powder and reflux-extracted three times with 10 L of 95% ethanol for 1 h per extraction. The combined filtrate was concentrated under reduced pressure to yield 437 g of the total crude extract.

The total extract was suspended in distilled water and sequentially fractionated with petroleum ether, dichloromethane, and ethyl acetate, with three extractions for each solvent. The ethyl acetate fraction was collected, concentrated under reduced pressure, and dried. The resulting residue was dissolved in 95% ethanol and further purified using a D101 macroporous adsorption resin column (5.5 cm × 50 cm).

Gradient elution was carried out using water–ethanol mixtures with ethanol concentrations of 0%, 30%, 50%, 70%. Each eluted fraction was concentrated and dried under reduced pressure. Finally, the 70% ethanol eluate was collected and used for subsequent experiments.

*A. tomentosa* saponin A was used as the reference standard to prepare a series of standard solutions. The absorbance was detected using a UV-Vis spectrophotometer (Hitachi, UH5300, Tokyo, Japan) for standard curve establishment, and the steroidal saponin content of the sample was quantified accordingly. Detailed experimental procedures are provided in the Supplementary Materials.

Subsequently, UPLC-MS/MS analysis was implemented to characterize the holistic chemical composition of EA. Briefly, 1.0 g of EA was mixed with 40 mL 80% aqueous methanol in a 50 mL centrifuge tube, followed by ultrasonic extraction for 30 min. Thereafter, 1 mL of the homogenized suspension was transferred into a 1.5 mL microcentrifuge tube and centrifuged at 12,000 rpm for 10 min at 4 °C. A total of 100 μL supernatant was collected and transferred into a chromatographic vial for subsequent UHPLC-MS/MS detection.

Chromatographic separation was performed on a Vanquish Flex UHPLC system (Thermo Fisher Scientific, Waltham, MA, USA) coupled with an ACQUITY UPLC HSS T3 column (2.1 mm × 100 mm, 1.7 μm, Waters Corporation, Milford, MA, USA). The mobile phase consisted of 0.1% formic acid aqueous solution (Phase A) and pure acetonitrile (Phase B). The flow rate was set at 0.3 mL/min, the column temperature was maintained at 40 °C, and the injection volume was 6.0 μL. The gradient elution procedure was detailed in Table 2.

Mass spectrometric detection was conducted using a Q Exactive hybrid quadrupole-orbitrap mass spectrometer (Thermo Fisher Scientific, Waltham, MA, USA) equipped with a HESI-II electrospray ionization probe. Nitrogen was adopted as the sheath gas, auxiliary gas and collision gas. Key ion source parameters: spray voltage, 3.7 kV (positive ion mode) and 3.5 kV (negative ion mode); heated capillary temperature, 320 °C; sheath gas pressure, 30 psi; auxiliary gas pressure, 10 psi; desolvation gas temperature, 300 °C; collision gas pressure, 1.5 mTorr.

All mass spectral data were acquired under the Full scan/data-dependent secondary mass spectrometry (Full scan/dd-MS^2^) mode, with the *m*/*z* scanning range set from 100 to 1500. For full-scan acquisition: mass resolution, 7000; automatic gain control (AGC) target, 1 × 10^6^; maximum injection time, 50 ms. For dd-MS^2^ acquisition: mass resolution, 17,500; AGC target, 1 × 10^5^; maximum injection time, 50 ms; top 10 intensity-dependent precursor ions for fragmentation; isolation window, *m*/*z* 2; stepped normalized collision energy, 10/30/60 V; intensity threshold, 1 × 10^5^.

Raw UHPLC-MS/MS data were processed using Progenesis QI 3.0 software (Waters Corporation, Milford, MA, USA), including raw data import, feature peak picking and adduct ion deconvolution. Metabolite identification was comprehensively validated via multi-dimensional matching criteria, including reference standard retention time deviation, precursor ion mass error, fragment ion matching score, isotopic distribution characteristics and characteristic peak area. Compound annotation was achieved based on the TCM Pro 2.0 database (Beijing Hexin Technology Co., Ltd., Beijing, China), together with literature-reported datasets and public online metabolomic databases.

### 4.2. Protective Effects of EA on HK-2 Cells Against COM Crystal-Induced Injury

HK-2 cells were incubated with different concentrations of EA for 24 h. Cell viability was detected via the Cell Counting Kit-8 (CCK-8, Cat. MK001B, Biomiky, Biotechnology Co., Ltd., Nanjing, Jiangsu, China), and non-cytotoxic concentrations were selected for subsequent experiments.

Cells were seeded in 96-well plates and cultured to 80% confluence. To construct an in vitro nephrolithiasis injury model, the culture medium was replaced with fresh medium containing 200 μg/mL COM crystals. For drug intervention, EA was added to the treatment groups at final concentrations of 3.13, 6.25, and 12.5 μg/mL, followed by further incubation for 24 h.

Subsequently, CCK-8 solution was added to each well, and after incubation for 45 min, the optical density (OD) at 450 nm was measured using a microplate reader. Cell viability was calculated according to the following formula: Cell viability (%) = [A(treated) − A(blank)]/[A(untreated) − A(blank)] × 100%.

### 4.3. Effects of EA on Intracellular ROS Generation and Apoptosis in COM Crystal-Stimulated HK-2 Cells

For intracellular reactive oxygen species (ROS) detection and apoptosis analysis, HK-2 cells were seeded in 6-well plates and subjected to the aforementioned treatments.

Intracellular ROS levels were detected using a commercial assay kit. Briefly, following 24 h of treatment, the cells were incubated with 200 μL of 10 μmol/L DCFH-DA working solution at 37 °C for 20 min in the dark. After washing three times with DMEM, the cells were observed and photographed under a fluorescence microscope. The fluorescence intensity was quantified using ImageJ 1.54f software, with four randomly selected fields analyzed per sample.

For apoptosis analysis, the culture medium was removed, and cells were rinsed with PBS. Subsequently, the cells were stained with 195 μL binding buffer containing 5 μL Annexin V-FITC and 10 μL propidium iodide (PI) staining solution. After incubation at room temperature for 20 min in the dark, cellular apoptosis was observed and photographed under a fluorescence microscope within 1 h.

### 4.4. Effects of EA on the Expression of PERK/ATF4/CHOP Signaling Pathway and Apoptosis-Related Proteins in HK-2 Cells

HK-2 cell modeling and EA administration were performed in accordance with the aforementioned procedures. For additional intervention treatments, 4-PBA (Cat. No. C23PD274A, Puxitang, China) and TM (Cat. No. A06HS183037, Yuanye Bio-Technology, Shanghai, China) were supplemented to the corresponding groups, with final concentrations of 164.2 µg/mL and 84 µg/mL, respectively.

After treatment, the cells were washed twice with PBS and lysed using cell lysis buffer. Cell lysates were collected into enzyme-free EP tubes and homogenized. Following centrifugation at 12,000 rpm for 10 min at 4 °C, the supernatant was harvested. The protein concentration was quantified via the BCA protein assay kit (Cat. No. 091923240531, Beyotime Biotechnology, Haimen, Jiangsu Province, China), and all protein samples were adjusted to a consistent concentration for subsequent experiments.

Loading buffer was added to the protein samples, and the mixtures were heated in a 100 °C water bath for 10 min. Protein separation was performed via SDS-PAGE, followed by wet transfer into PVDF membranes (Cat. No. RTKA8928E, Merck, KGaA, Darmstadt, Germany). The membranes were blocked with skimmed milk and incubated with primary antibodies overnight at 4 °C. After washing three times with TBST, the membranes were incubated with secondary antibodies for 2 h at room temperature.

Protein bands were visualized using ECL chemiluminescent reagent (Cat. No. 111423240530, Beyotime Biotechnology, Haimen, Jiangsu Province, China) and a gel imaging system (Tanon 5200 Multi, Shanghai Tanon Life Science Co., Ltd., Shanghai, China). The gray values of the bands were analyzed using ImageJ 1.54f software (National Institutes of Health, USA) and normalized to β-actin. The relative expression levels of BIP, p-PERK, ATF4, CHOP, Cleaved-casapase3, Bax and Bcl-2 (Lot No. 3f54342, 3g55321, 65o3917, DF6008, 15z0096, 44q6915, 11o9905, Affinity Biosciences Group Ltd, Changzhou, Jiangsu, China) were subsequently calculated.

### 4.5. Animal Grouping, Drug Administration, and Sample Collection

Forty-eight 8-week-old male C57BL/6 mice were purchased from SPF Biotechnology Co., Ltd. (Beijing, China), with the animal quality certificate number 110324241106493028 and laboratory animal use license SYXK (Yun) 2024-0003. All animal experimental protocols were approved by the Animal Ethics Committee of Yunnan Branch of Institute of Medicinal Plant Development, Chinese Academy of Medical Sciences & Peking Union Medical College (Approval No. 20241104033). Mice were housed in a specific pathogen-free laboratory at 20 ± 2 °C with relative humidity of 55 ± 5%, under a 12 h light/dark cycle. Animals had free access to standard chow and drinking water.

The mouse dosages were calculated based on extract yield and body surface area ratio, equivalent to daily crude drug intakes of 7.5 g, 15 g and 30 g in humans, which conforms to the clinical routine dosage range. 4-PBA was prepared following published protocols, with the dosage adjusted based on preliminary experimental data [42].

The mice were acclimatized for one week and randomly divided into six groups (n = 8 per group): control group, glyoxylate (Gly) group, low-dose EA group (EA-L, 2.6 mg/kg), medium-dose EA group (EA-M, 5.2 mg/kg), high-dose EA group (EA-H, 10.4 mg/kg), and 4-PBA (250 mg/kg) group.

All animals were allowed free access to food and drinking water. The drugs were suspended in 0.5% sodium carboxymethyl cellulose solution and administered intragastrically. Mice were pretreated with corresponding drugs for 4 days prior to model establishment. Except for the control group, all mice received daily intraperitoneal injection of 70 mg/kg glyoxylate (Cat. No. 0000243359, Sigma-Aldrich, St. Louis, MO, USA) for 10 consecutive days to induce calcium oxalate nephrolithiasis.

At the end of the experiment, mice were anesthetized, and blood samples were collected in anticoagulant tubes. Serum creatinine (Scr) and blood urea nitrogen (BUN) levels were measured using an automatic biochemical analyzer (IDEXX VetTest 8008, IDEXX Laboratories Inc., Westbrook, ME, USA).

Both kidneys were harvested immediately. The right kidney was used for histopathological examination and TUNEL staining, while the left kidney was prepared for Western blot analysis in accordance with the procedures described in Section 4.4.

### 4.6. Histomorphological Observation

The right kidney tissues were fixed in 4% paraformaldehyde (Cat. No. 23333866, Biosharp, Hefei, Anhui, China) for 72 h, dehydrated, embedded in paraffin, and serially sectioned. Hematoxylin-eosin (HE) staining (Hematoxylin-eosin staining kit, Cat. No. 2309004, Solarbio Science & Technology Co., Ltd., Beijing, China) and TUNEL fluorescence staining (TUNEL fluorescence staining kit, Cat. No. 7E1650K4, Vazyme Biotech Co., Ltd., Nanjing, Jiangsu, China) were conducted in strict accordance with the manufacturer’s instructions.

Following TUNEL staining, the tissue sections were mounted and observed under a microscope for image acquisition. The number of TUNEL-positive cells and total nucleated cells was quantified using ImageJ 1.54f software, and the proportion of TUNEL-positive apoptotic cells was calculated.

### 4.7. Statistical Analysis

All data were statistically analyzed using GraphPad Prism 9.5 software (GraphPad Software, San Diego, CA, USA) and expressed as the mean ± standard deviation (SD). Homogeneity of variance and normality tests were performed first. One-way ANOVA was used for data with normal distribution and homogeneous variance, while the Kruskal–Wallis test was applied for non-normal or heteroscedastic data. A *p*-value < 0.05 was considered statistically significant.

## 5. Conclusions

Collectively, the present study provides comprehensive evidence that EA confer robust preventive effects against calcium oxalate nephrolithiasis through multi-targeted protective actions in both in vitro and in vivo models. EA effectively mitigated COM crystal-triggered injury in renal tubular epithelial cells, attenuated intracellular reactive oxygen species overproduction, and suppressed aberrant apoptotic cell death. In the Gly-induced murine nephrolithiasis model, EA treatment markedly reduced renal calcium oxalate crystal deposition, ameliorated histopathological deterioration of renal tissues, and restored impaired renal function, as evidenced by decreased serum creatinine and blood urea nitrogen levels. Mechanistically, EA significantly blunted the activation of the PERK/ATF4/CHOP signaling cascade, thereby alleviating endoplasmic reticulum stress and abrogating endoplasmic reticulum stress-mediated renal cell apoptosis. These findings elucidate the core molecular mechanism responsible for the anti-nephrolithiasis efficacy of EA and validate the traditional clinical application of A. tomentosa for the prevention and management of renal calculi. Importantly, this study highlights that the steroidal saponin-enriched extract of *A. tomentosa* serves as a promising natural candidate for the prophylactic intervention of nephrolithiasis, establishing a solid scientific basis for the further development and sustainable utilization of this valuable ethnic medicinal resource.

## Figures and Tables

**Table 1:** Compounds information.

NO.	Retention Time (Min)	Compound	Chemical Class	Molecular Formula	Chemical Structure	MS^1^ *m*/*z*	Primary Fragment Ions (ESI-MSn) *m*/*z* (%)
1	0.91	2-[(2R,3R,4S,5S,6R)-3,4,5-trihydroxy-6-(hydroxymethyl)oxan-2-yl]-1,2-oxazol-5-one	Isoxazolinone glycoside	C_9_H_13_NO_7_	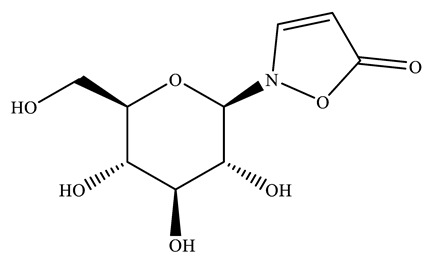	292.0673	112.038, 59.0113, 82.0272, 292.068, 71.0113, 124.0381, 126.0174, 84.0428, 89.022, 57.0321
2	4.21	3,4-dihydroxybenzoic acid	Phenolic acid	C_7_H_6_O_4_	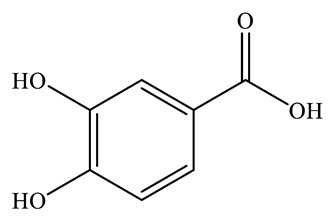	155.0338	111.0445, 93.0343, 65.0399, 137.0232, 155.0337, 109.0289, 154.0497, 137.0469
3	4.57	Koaburaside	Phenolic glycoside	C_14_H_20_O_9_	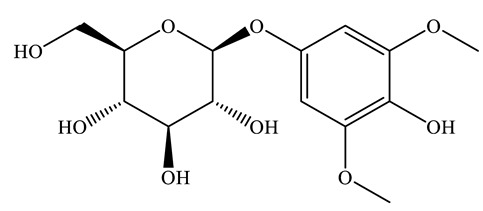	335.0995	171.065, 139.0389, 85.0294, 111.0445, 267.99, 97.0292, 137.0232, 127.0392, 69.0349, 249.9792
4	5.27	Apobcoroside	Protein/Peptide	C_13_H_16_O_8_	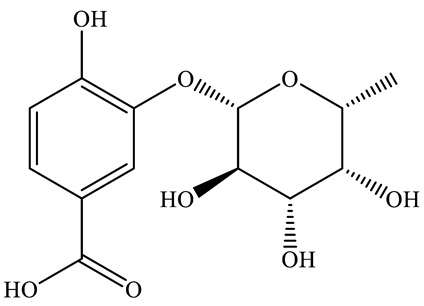	283.0809	217.0493, 121.0286, 151.0389, 55.0193, 247.0593, 105.0704, 127.039, 283.0812, 242.0105, 266.1386
5	7.05	Catechin	Flavonoid	C_15_H_14_O_6_	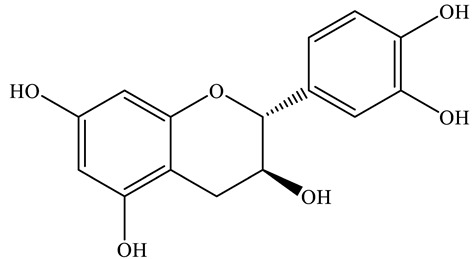	291.0857	123.0443, 273.0751
6	12.32	1-azeloyl-rac-glycer	Monoacylglycerol	C_12_H_22_O_6_	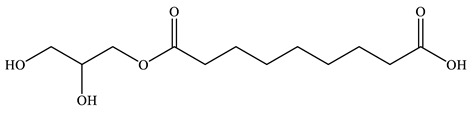	245.1380	227.1275, 125.0963, 97.1019, 55.0557, 189.1122, 67.0555, 79.0553, 107.0861, 83.0865, 81.071
7	13.80	(+)-lyoniresinol	Lignan	C_22_H_28_O_8_	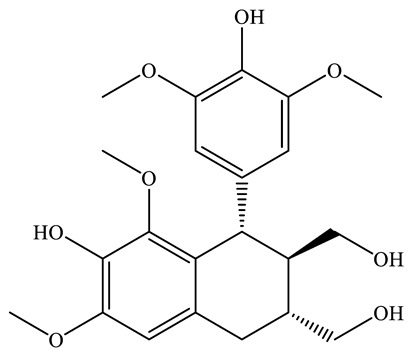	403.1746	359.1483, 167.0702, 161.0598, 181.0495, 137.0596, 233.0805, 387.1411, 107.0496, 203.0692, 250.0695
8	14.31	Helonioside A	Steroidal saponin	C_32_H_38_O_17_	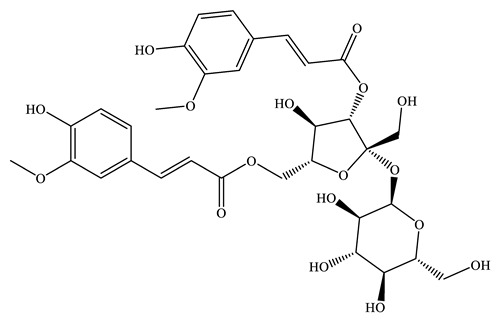	693.2041	175.0384, 243.1234, 160.0146, 693.2043, 225.1123, 193.0493, 207.1016, 134.0351, 132.0195, 99.0063
9	15.19	Helonioside B	Steroidal saponin	C_34_H_40_O_18_	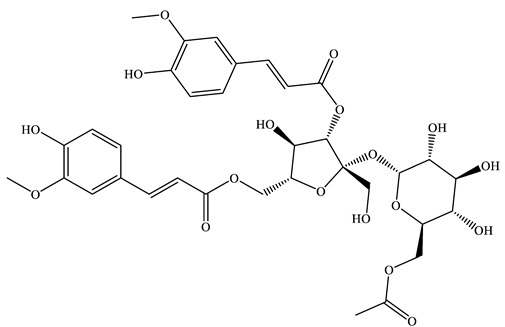	735.2155	175.0384, 160.0147, 735.2151, 193.0493, 134.0351, 132.0195
10	15.24	Quercetin	Flavonoid	C_15_H_10_O_7_	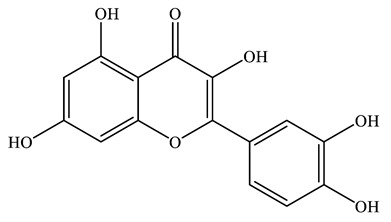	303.0493	303.0497, 153.0181, 229.0495, 137.0234
11	16.01	Smilaside B	Phenylpropanoid glycoside	C_34_H_40_O_18_	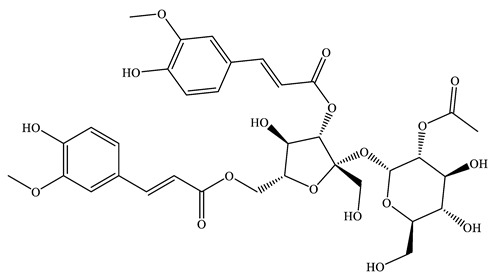	735.2152	175.0383, 160.0146, 735.2153, 193.0492, 134.0351, 132.0194
12	17.47	Fulgidic acid	Depside	C_18_H_32_O_5_	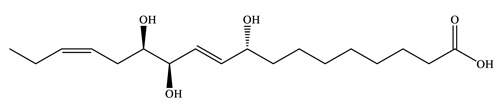	327.2176	373.2224, 201.1119, 139.1107, 371.1138, 268.1092, 171.1011, 183.101, 327.1223, 75.006, 267.1028
13	17.92	Smiglaside C	Phenylpropanoid glycoside	C_38_H_44_O_20_	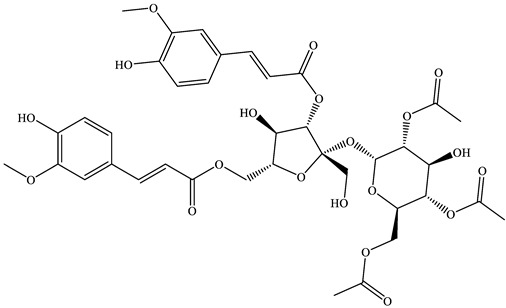	838.2743	177.0545, 145.0283, 515.1547, 557.1649, 117.0339, 127.0392, 109.0289
14	17.99	(9s,10e,12s,13s)-9,12,13-trihydroxy-10-octadecenoic acid	Hydroxy fatty acid	C_18_H_34_O_5_	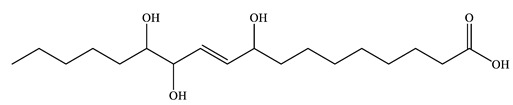	295.2261	295.2264, 277.2157, 67.0555, 81.071, 55.0557, 93.0708, 95.0863, 109.1017, 69.0712, 107.0862
15	19.04	2,3-seco-Sond15erianol	Diterpenoid	C_20_H_26_O_5_	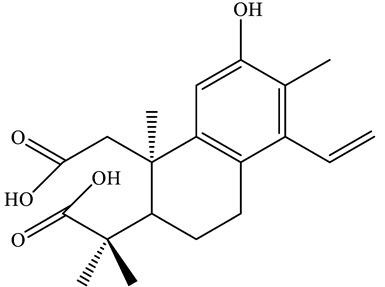	345.1706	345.1707, 283.1704, 267.1393, 301.1809, 199.1116, 257.1911
16	20.01	2α,3β,23-trihydroxyolean-12-en-28-oic acid	Triterpenoid	C_30_H_48_O_5_	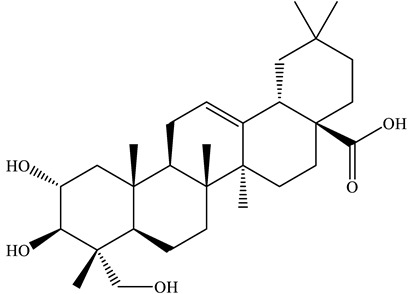	533.3486	487.3454, 533.3423, 116.926, 61.9857, 485.3286
17	20.33	Aspidatasin B	Lignan glycoside	C_33_H_46_O_8_	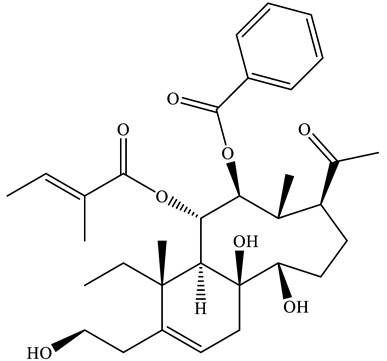	567.2943	123.0443, 151.0389, 415.2467, 277.216, 163.0389, 147.0441, 93.0707, 445.186, 81.0709, 67.0555
18	20.90	Aspidatasin A	Lignan glycoside	C_32_H_48_O_8_	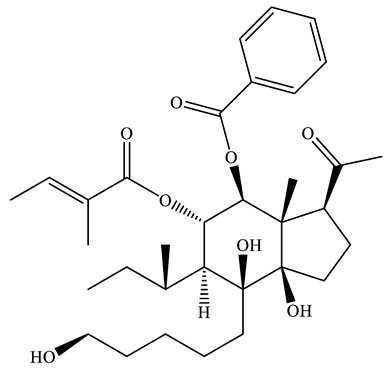	569.3099	123.0443, 151.0389, 417.2635, 445.1852, 163.0388, 147.044, 247.1327, 293.1378, 279.2318, 67.0555
19	21.84	Asperphenamate	Phenylpropanoid amide alkaloid	C_32_H_30_N_2_O_4_	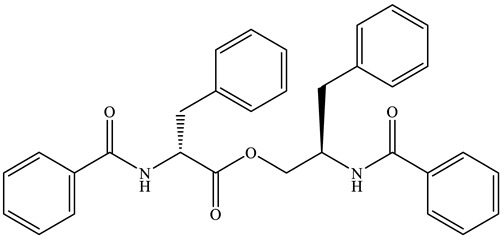	507.2268	105.0341, 238.1222, 117.0702, 507.228, 256.1328, 224.1066, 91.055, 122.0603
20	24.76	(24s)-24-ethylcholesta-3β,5α,6β-triol	Sterol	C_29_H_52_O_3_	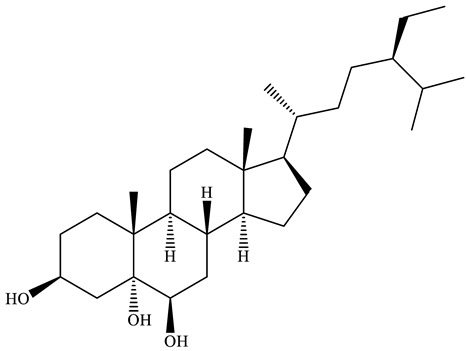	413.3768	413.3799, 81.0709, 95.0863, 395.3685, 411.3657, 69.0712, 159.1166, 107.086, 105.0705, 93.0707
21	25.92	Palmitic acid	Fatty acid	C_16_H_32_O_2_	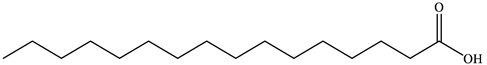	255.2326	255.2327

**Table 2:** Elution gradient.

Time (min)	Mobile Phase	
	A (v%)	B (v%)
0	98	2
1.0	98	2
14.0	70	30
25.0	0	100
28.0	0	100
28.1	98	2
30.0	98	2

## Data Availability

The original contributions presented in this study are included in the article. Further inquiries can be directed to the corresponding authors.

## Supplementary Materials

The supporting information can be downloaded at https://www.mdpi.com/article/10.3390/ph19071049/s1, Figure S1: Standard curve of Obcordata A; Figure S2: TUNEL fluorescence staining to detect the apoptosis level of mouse kidney tissues. (A) Representative TUNEL fluorescence staining images of kidneys from different groups (200×). (B) EA reduces the apoptosis rate in renal tissue of kidney stone model mice. Note: Compared with the con group, ^##^ indicates *p* < 0.01; compared with the COM group, ** indicates *p* < 0.01.

## Abbreviations

The following abbreviations are used in this manuscript:

EA	The extracts of *Aspidopterys obcordata*
ROS	Reactive oxygen species
Scr	Serum creatinine
BUN	Blood urea nitrogen
ERS	Endoplasmic reticulum stress
TM	Tunicamycin
CaOx	Calcium oxalate
EG	Ethylene glycol
Grp78	Glucose-regulated protein 78
eIF2α	Eukaryotic translation initiation factor 2α
p-PERK	Phosphorylated PERK
COM	Calcium oxalate monohydrate
OD	Optical density
PI	Propidium iodide
Gly	Glyoxylate
HE	Hematoxylin-eosin
SD	Standard deviation
