# Supplementary material for: Extracts of Aspidopterys tomentosa Attenuate Nephrolithiasis via Inhibiting Endoplasmic Reticulum Stress
Source: Pharmaceuticals (Basel). 2026 Jul 7;19(7):1049. doi: 10.3390/ph19071049 (PMC13415603; doi:10.3390/ph19071049)
Supplement: Supplementary file 1 [file pharmaceuticals-19-01049-s001.zip › pharmaceuticals-4318452-supplementary.pdf]

1. Determination of Steroidal Saponins Content in EA

Accurately weigh 5 mg of self-prepared Obcordata A reference standard with purity higher than 98%, dissolve it in methanol and make up the volume to 50 mL. Precisely pipette 1 mL of the solution and heat in a water bath until methanol is fully evaporated. After cooling to room temperature, add 2 mL glacial acetic acid, 1 mL freshly prepared 5% vanillin-glacial acetic acid chromogenic solution and 1 mL perchloric acid in sequence. Vortex the mixture evenly and heat in a water bath at 60 °C for 20 min. Upon cooling, ultraviolet spectrophotometric scanning was conducted within 200–700 nm. The maximum absorption peak of Obcordata A was detected at 325 nm.

Reference solutions with different concentrations were prepared. A standard curve was plotted with the concentration of reference substance as the abscissa and absorbance as the ordinate, and the regression equation was obtained via linear regression analysis.

An appropriate amount of sample was weighed, dissolved with methanol and diluted to constant volume. After the same chromogenic reaction, the absorbance was measured and the content of steroidal saponins was calculated.

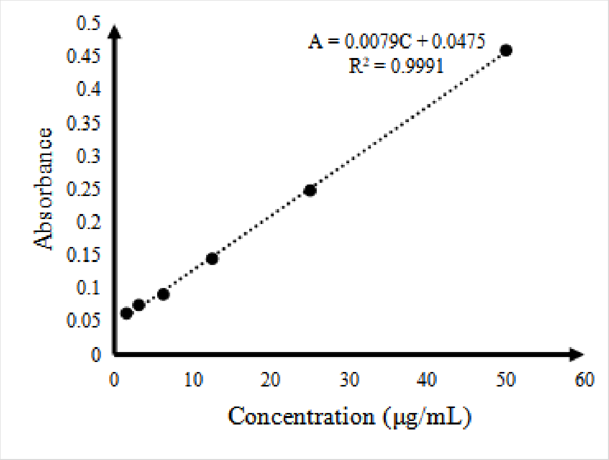

Figure S1 Standard curve of Obcordata A

2. EA reduces the apoptosis rate in renal tissue of nephrolithiasis model mice.

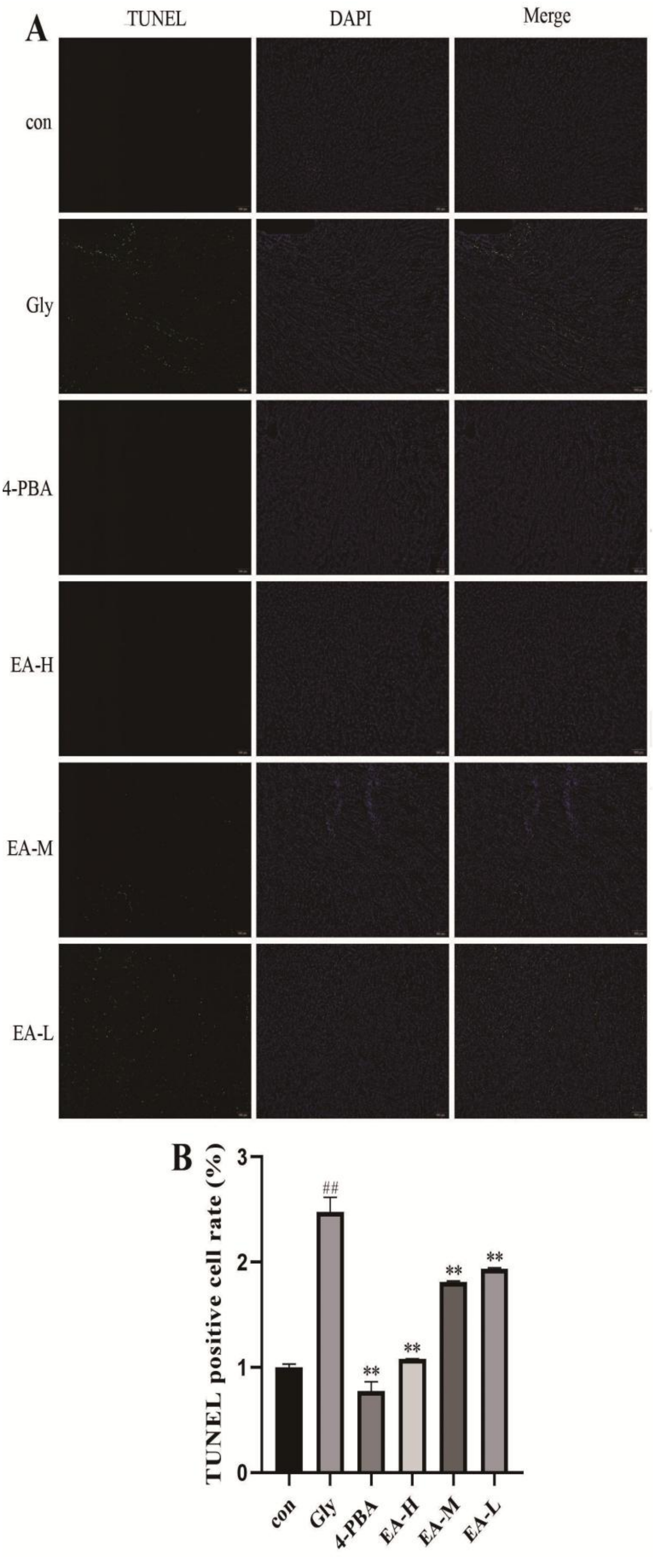
A
TUNEL
DAPI
Merge
con
Gly
4-PBA
EA-H
EA-M
EA-L
B
TUNEL positive cell rate (%)
0
1
2
3
##
**
**
**
**
**
con
Gly
4-PBA
EA-H
EA-M
EA-L

Figure S2 TUNEL fluorescence staining to detect the apoptosis level of mouse kidney tissues. (A)Representative TUNEL fluorescence staining images of kidneys from different groups (200×). (B)EA reduces the apoptosis rate in renal tissue of kidney stone model mice. Note: Compared with the con group, ##indicates $p < 0.01$; compared with the COM group, **indicates $p < 0.01$.
